# Exploring the Transport Path of Oceanic Microplastics in the Atmosphere

**DOI:** 10.1021/acs.est.4c03216

**Published:** 2024-07-30

**Authors:** Silvia Bucci, Camille Richon, Lucie Bakels

**Affiliations:** † Department of Meteorology and Geophysics, University of Vienna, Universitätsring 1, Vienna 1010, Austria; ‡ Laboratoire d’Océanographie et du Climat: Expérimentations et Approches Numériques, Institut Pierre Simon Laplace (LOCEAN-IPSL), Sorbonne Université, CNRS, IRD, MNHN, 75005 Paris, France; ¶ Laboratoire d’Océanographie Physique et Spatiale (LOPS), UMR 197 CNRS/IFREMER/IRD/UBO, Institut Universitaire Européen de la Mer, Plouzané 29280, France

**Keywords:** ocean pollution, atmospheric transport, emissions, deposition, stratosphere, sea spray

## Abstract

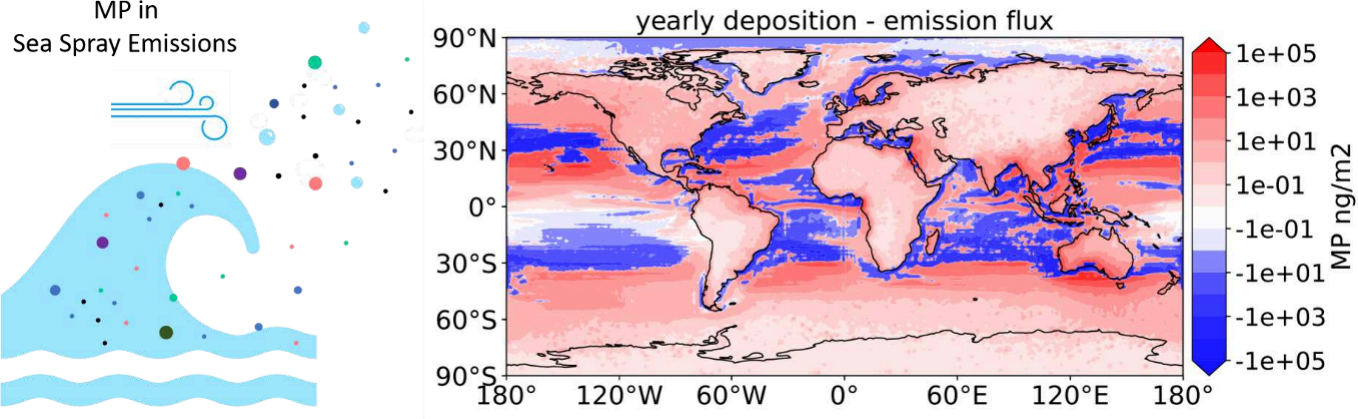

Microplastics (MP) have been recognized as an emerging atmospheric pollutant, yet uncertainties persist in their emissions and concentrations. With a bottom-up approach, we estimate 6-hourly MP fluxes at the ocean-atmosphere interface, using as an input the monthly ocean surface MP concentrations simulated by the global oceanic model (NEMO/PISCES-PLASTIC, Nucleus for European Modeling of the Ocean, Pelagic Interaction Scheme for Carbon and Ecosystem Studies), a size distribution estimate for the MP in the micrometer range, and a sea salt emission scheme. The atmospheric dispersion is then simulated with the Lagrangian model FLEXPART. We identify hotspot sources in the tropical regions and highlight the seasonal variability of emissions, atmospheric concentrations, and deposition fluxes both on land and ocean surfaces. Due to the variability of MP concentration during the year, the MP flux from the sea surface appears to follow a seasonality opposite to that of sea salt aerosol emissions. The comparison with existing observations of MP in the marine atmosphere suggests an underestimation of one to 2 orders of magnitude in our current knowledge of the MP in the oceans’ surface. In addition, we show that the MP in the micrometer range is transported efficiently around the globe and can penetrate and linger in the stratosphere over time scales of months. The interaction of these particles with the chemistry and physics of the atmosphere is still mostly unknown and deserves to be further investigated.

## Abstract 2

We investigate the atmospheric emission of microplastic from the ocean and describe their pathways of transport and deposition over a one year period.

## Introduction

An increasing number of studies revealed that plastic pollution, in particular in the form of microplastic particles (MP), can be found in any environmental compartment.^1^ MP presence in the ocean has been long recognized as a pollution threat to the marine environment.^2−6^ Due to the low density of some of the plastic polymers such as polyethylene (PE) and polypropylene (PP) (0.86–0.96 *g* · *cm*^–3^), MP can accumulate at the seawater surface for a total global estimate up to 51.2 trillion pieces of MP (for diameters below 200 mm) and a total mass up to 236 thousand metric tons.^4^

Processes such as wave breaking and bubble bursting can inject marine MP in the atmosphere along with sea spray:^7−10^ Allen et al., during a dedicated campaign along the French Atlantic coast, identified MP particles and sea spray droplets in the marine boundary layer air, measuring an average concentration of 2.9 MP particles *m*^–3^ onshore; Trainic et al. collected ambient aerosol samples in the North Atlantic Ocean, finding airborne PE, PP and polystyrene (PS) particles as small as 5 *μm*, identifying their source in the ocean by back-trajectories analysis; Ferrero et al. collected both airborne and marine MP over the Baltic Sea region and found similar concentrations and compositions for both the suspended and surface water particles, suggesting the possible exchange between marine and atmospheric compartments.

A correct assessment of the emissions and dispersion of plastic particles is a question of environmental significance, as MP transported in the atmosphere can have adverse effects on the ecosystem and human health. In particular, inhalation has been shown to be the most dangerous exposure route for humans.^11,12^ In addition to the well-known mechanical harms caused by plastic pollution (e.g., by ingestion and mechanical stress^13^), MP has been demonstrated to have inflammatory effects on cells due to the release of reactive oxygen species.^14−16^ MP, can also easily adsorb organic and inorganic pollutants, therefore acting as a vector for toxic and carcinogenic pollutants including heavy metals, polycyclic aromatic hydrocarbons, persistent organic pollutants and pathogens.^17−21^ Furthermore, the MP that enters the atmosphere from the ocean surface can act as a vector for viruses, bacteria and other organic compounds, as biofilms rapidly develop on MP surfaces in aquatic habitats.^22^ The biofilm can alter the physical and chemical properties of microplastics, potentially affecting their hygroscopicity, toxicity, and ability to transport pollutants.^22,23^ Atmospheric transport and deposition of MP can represent a route of widespread exposure for humans as well as for the ecosystem: it can affect marine life along pathways that go further beyond the typical ocean currents and reach terrestrial environments far away from populated regions. The transport of MP has been demonstrated to also affect atmospheric layers above the planetary boundary layer (e.g., MP presence has been observed at Pic du Midi at 2877 m altitude by Allen et al.^24^ and up to 3500 m during the aircraft measurements of González-Pleiter et al.)^25^ and the atmospheric advection in the free troposphere has been hypothesized as a main driver for the MP deposition over remote areas (e.g.^26−28^). As any other aerosol, MP has the potential to alter the climate through interaction with solar radiation,^29^ by acting as nuclei for the formation of liquid cloud droplets and ice crystals,^30^ and contributing to variations of snow albedo.^31,32^

A few recent studies tried to quantify the global flux of MP between the sea surface and air from laboratory experiments (0.72–4.13 tons yr-1 for Dp< 10 *μm*, Harb et al.^33^ and 20,000–7,400,000 tons yr^–1^ for Dp < 280 *μm*, Shaw et al.^34^). These papers demonstrated the capability of the ocean to eject plastic particles by bubble-bursting processes, but do not investigate the subsequent transport in the atmosphere and the redeposition on the sea surface. Other studies computed the global fluxes based on extrapolation from collected data or from inverse modeling.^7,8,35^ Yang et al. estimated global average marine emissions to be 773 (∼30–1515) *ton* · *yr*^–1^, based on upscaling sea spray aerosols (SSA) values, provided by GEOS-Chem simulations, and laboratory studies of MP emissions by SSA. Other studies tried to assess the global fluxes from ocean surface by inverse modeling,^27,35,36^ estimating respectively 8600 *ton* · *yr*^–1^, 8900 ± 3500 *ton* · *yr*^–1^ and 418000 ± 201000 *ton* · *yr*^–1^, or by an upscale of marine boundary layer observations,^7^ giving estimates of 136000 *ton* · *yr*^–1^ blowing ashore.

Most of these studies are based on oversimplified assumptions, such as the direct linear correlation of sea spray atmospheric flux with the emissions of MP (e.g.^33,36^) which may lead to an overestimate of the flux. In addition, all of these studies assume a constant concentration in time of the sea surface MP and do not consider a realistic size distribution of the MP from the sea (with the exception of Shaw et al. that includes a power law representation for the fragmented microplastic). The inversion modeling approaches (e.g.^8,35,36^) may also bring along large uncertainties, mainly due to the extrapolation from limited observations. A more realistic description and quantification of the 3-dimensional distribution of MP in the atmosphere is crucial to have a correct assessment of the climatic risks related to the increasing presence of those particles in the atmosphere.

To contribute to addressing these open questions, we provide an analysis of the atmospheric pathways of MP generated by the sea spray fluxes. We therefore use a bottom-up approach, aiming at including the parameters that are most likely to affect atmospheric transport. We first estimate a size distribution of the MP on the ocean surface in the ranges that are relevant for atmospheric transport. We then consider the temporal and spatial evolution of the MP load. We do this by modeling the monthly variability of MP surface concentrations and linking these with the 6-hourly variability of sea spray emissions across the globe. This way, we generate a global database of oceanic MP emissions over a full year with a 6-h resolution. Linking these emissions with a 1-year long global atmospheric Lagrangian simulation, we investigate the impact of oceanic sources on the atmospheric concentrations of MP. We focus our results on the analysis of the horizontal and vertical advection of injected MP, as well as their deposition fluxes on land and ocean surfaces and their seasonality.

## Methods

The modeling approach adopted in this study consists of 4 steps: 1) Quantification of the monthly MP load at the ocean surface from the NEMO/PISCES-PLASTIC model, 2) specification of the MP size distribution in the micrometer range, 3) application of a sea spray scheme for quantifying the MP emission, 4) simulations of global atmospheric MP dispersion with FLEXPART.

### MP Load at the Ocean Surface

To have a realistic estimate of the spatial distribution of MP at the ocean surface, and its possible seasonal evolution, we exploit the NEMO-PISCES (Nucleus for European Modeling of the Ocean, Pelagic Interaction Scheme for Carbon and Ecosystem Studies) general circulation model, with the specific configuration named PISCES-PLASTIC,^37^ as it provides not only a realistic estimate of the spatial distribution of MP at the ocean surface but also its monthly variability, thereby capturing the seasonal variability that is usually not accounted for in other models of marine MP. The model, described in Richon et al., has a horizontal resolution of 2° with 31 vertical levels (10 levels in the first 100 m) and provides a monthly estimate of the mass concentration of microplastic transported by rivers^38^ that is passively transported by ocean currents. The MP concentration in the upper level of the NEMO/PISCES-PLASTIC model (average of the first 10 m from the water surface) matches with the highest estimates of the work of Sebille et al.^4^ (see also the comparison discussed in Richon et al.^37^). The MP particles represented in this model correspond to 3 densities (floating, neutral and sinking MP) with no specific shape or size. The ocean model was run using climatological physical and biogeochemical forcings (i.e., wind, currents, sea surface temperature, sea surface salinity, freshwater and nutrient fluxes), similarly to Richon et al., Aumont et al.^39^ The model follows the three-dimensional pathways of ocean currents and therefore takes into account possible seasonal sinks of MP from the ocean surface by downwelling motions. For the purpose of this study, we use the floating particles (representing a large fraction of the most common polymers found in the ocean, such as polypropylene, polystyrene and high- and low-density polyethylene,^40^) on the upper model level (0–10 m), as we expect that most of the particles that are injected in the atmosphere are the ones floating on the surface. Therefore, also for the atmospheric transport simulations, we assume a particle density of 1010 *kg* · *m*^–3^, slightly less than the average ocean water density (1020 *kg* · *m*^–3^).^41^

### Size Distribution of MP in Water

One of the big challenges in the determination of MP characterization in the environment is the identification of the size distribution. The particles with diameters below a few tens of *μm* in sea waters are especially complicated to identify due to the technical limitations of the sampling method for MPs. One of the most common sampling methods is the use of plankton sampling nets, which typically have a mesh size of around 300 *μm*.^42^ However, the smaller size range (diameter Dp < 60 μm) is the focus of this study, as small particles are more likely to be transported over long distances in the atmosphere and do not immediately settle back at the surface by gravitation. The MP surface concentration from NEMO/PISCES-PLASTIC matches with the highest estimates of the work of Sebille et al. (see Richon et al.). To obtain a size distribution of particles in the microplastic range, we start with the log-normal distribution (a possible simplification of the larger MP distribution in the ocean^43−45^) that fits the upper estimates of global mass and the global particle number count from Sebille et al. For a number of particles (MPn) of 51.2 trillions, we obtain a log-normal distribution with standard deviation σ = 0.66 and mean μ = 0.11, which has a similar shape to what was observed in other studies.^46,47^ We will assume this distribution to be representative for the MP larger than 300 *μm*. As we want to determine the number of small MP that are formed by fragmentation of bigger debris, we extend this size distribution assuming a power law behavior *N* = C*(*Dp*)^*n*^ (Figure S1a), where *N* is the total number of particles, *C* a constant and *n* = −3 ± 0.3, a scaling exponent that has been shown to be a good approximation for the fragmentation processes of aged marine MP.^48^ We compute *C* by minimizing the distance between the power law and the log-normal distribution previously estimated, obtaining C = 8.84e10 for *n* = −3, C = 6.945e10 for *n* = −2.7 and C = 1.19e11 for *n* = −3.3. More details are explained in section S1 and the resulting size distributions are shown in Figure S1b. With respect to the estimate of Sebille et al., we obtain with this approach a total number of particles at the surface increased by 1e9 trillion particles (around 105,026 tons) for *n* = −3.3, 1e8 trillion particles (around 51,712 tons) for *n* = −3 and 1e7 trillion particles (around 27,619 tons) for *n* = −2.7. To simulate the behavior of the particles resuspended in the atmosphere we will only use the size ranges covered by the resulting power law (i.e., between 1 and 60 μm). We used the *n*=-3 power law as a reference for our study, and *n* = −2.7 and *n* = −3.3 as boundaries for our uncertainties. For the atmospheric simulations, we will use five representative size bins for Dp: 1–5,5–10,10–25,25–50, and 50–60 *μm*, chosen to cover most of the coarse mode aerosol relevant for atmospheric transport while still discriminating between different lifetimes in the atmosphere. For this study, we decided to not include sizes smaller than 1 μm, as a further extrapolation of the size distribution would increase the uncertainties in the estimates. However, such particle sizes are not expected to represent a significant fraction of the emitted MP mass.^8,33^

### MP Atmospheric Emissions Carried by Sea Spray

**Figure 1:**
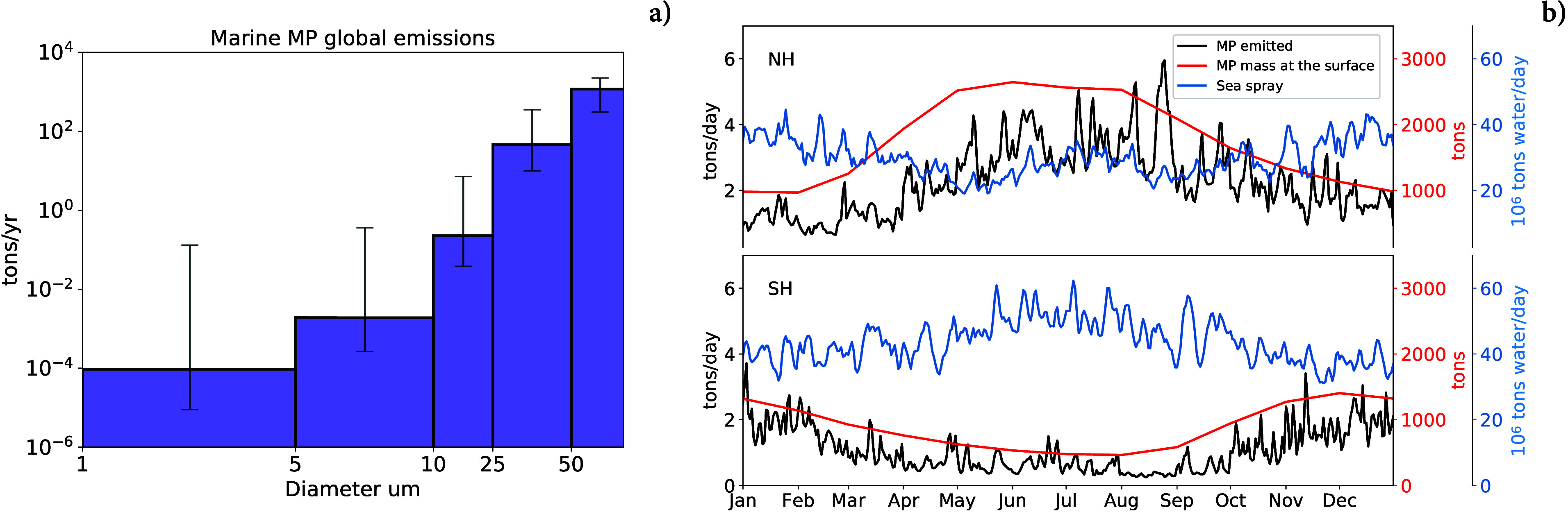
Panel a) Simulated yearly global emission of marine MP mass per size bin. The error bars represent the lower and higher estimates using respectively *n* = −2.7 and *n* = −3.3 as a power law exponent (See methods). Panel b) MP emissions, total MP mass floating at the surface, and sea spray mass, all for particles with Dp ≤ 60 *μm*, integrated over the Northern Hemisphere (upper plot) and the Southern Hemisphere (lower plot).

To estimate the intensity and variability of the sea spray emissions, we made use of the sea spray source function  defined in Grythe et al. This source function, depending on the wind speed at 10 m (*U*_10_) and the sea surface temperature (T), gives the flux of droplet number as a function of the diameter *D*_*p*_. When used as a source for the FLEXPART Lagrangian dispersion model, it was demonstrated to be a good emission scheme for comparing the simulated and the observed concentrations of sea salt.^49^ We produced an estimate of the global flux of sea spray at 1° × 1° and 6 hourly resolution, using as input the ERA5 reanalysis data for the year 2014, to be consistent with the input data of MP concentrations used to calculate the oceanic MP size distribution.^4^ The flux of water droplets is computed for each of the 5 diameter sizes highlighted in Figure 1. The assumption is that each droplet can carry only microplastic that can fit in its diameter. To obtain the mass flux of MP in the atmosphere, for each size bin we coupled the flux of emitted sea spray in units of droplet volume (*m*^3^) with the concentration of MP in seawater surface (kg · *m*^–3^). The MP mass flux (*MP*_*f*_) into to the atmosphere is given by
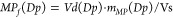
1where *Vd*(*Dp*) is the volume of sea spray carrying particles of dry diameter Dp, as computed from the method of Grythe et al., *m*_*MP*_(*Dp*) is the mass of MP from the uppermost NEMO-PISCES model layer and Vs is the total volume of seawater of the same layer (so that the ratio *m*_*MP*_(*Dp*)/(V s) represents the density of microplastic in the water close to the sea surface). The mass concentration for each size bin is computed starting from the values from NEMO/PISCES-PLASTIC model. As the model gives the MP concentration associated with particles Dp ≥ 300 *μm* we extend the corresponding mass to the smaller size bins down to Dp = 1 *μm*, using the size distribution of Figure S1.

### Global Atmospheric Transport and Deposition of Oceanic MP

The global simulations of atmospheric transport of oceanic MP are performed using the Lagrangian particle dispersion model FLEXPART (FLEXible PARTicle dispersion model,^50,51^) version 11 from Bakels et al.,^52^ driven by the global hourly meteorological data from ERA5 at a 0.5° × 0.5° horizontal resolution and 137 vertical levels up to 1 Pa. We perform a simulation for each of the five size ranges (1–5,5–10,10–25,25–50 and 50–60 *μm*), assuming that the particles are spherical, and releasing particles at intervals of 6 h for the whole 2014 year, starting from our 1° × 1° gridded oceanic MP emission estimate. The sea spray droplets are assumed to be released at 10 m above sea level, as done in Grythe et al. The simulation includes a two-month spin-up (starting from November 2013) and involves a total release of 60 million air parcels that are followed forward in time. The output is produced with a 1° × 1° horizontal resolution, 1 km vertical resolution, and 6 h temporal resolution. The fluxes of dry^50^ and wet deposition^53^ are also computed by FLEXPART in the same horizontal and time resolution.

### MP Scavenging Efficiency Sensitivity

MP particles in water can undergo aging processes that makes these particles highly hydrophilic and possibly able to act as ice or even cloud nuclei (e.g., by photochemical oxidation, sorption of macromolecules or trace soluble species, biological coating and/or oxidation^32,54^). We, therefore, hypothesized the emitted MP to be efficient cloud and ice scavenging particles, and we used the same scavenging features previously tested for sulfate aerosol in FLEXPART, as it is known to be an aerosol with high scavenging efficiencies, see Grythe et al. For completeness, we also test the sensitivity to various scavenging efficiencies, similarly as done in Evangeliou et al. The results of the sensitivity study on the scavenging properties are presented in section S2 of the Supporting Information.

## Results and Discussion

### MP Sea Spray Emissions

Our simulation results indicate that 1231 tons yr^–1^ (range: 320–5,383 tons yr^–1^) of MP particles (Dp < 60 *μm*) are emitted globally from the surface ocean to the atmosphere (see Figure 1). This estimate is significantly lower than what was found in other studies working with comparable size ranges (emissions of the order of 10^4^ – 10^6^ tons yr^–1^^7,35,36,55^) but in a similar order of magnitude as the analysis done by Yang et al. (30 to 1515 tons yr^–1^). We also obtained similar results to Harb et al. when we integrate our fluxes for particles in the same size range (Dp < 10 μm), giving 0.04–7.73 tons yr^–1^ compared to the 0.72–4.13 tons yr^–1^ reported by Harb et al. The seasonal variability of MP emissions is mostly determined by the ocean surface MP concentration (Figure 1). Despite the coldest months in both hemispheres being characterized by the strongest sea spray production, the highest MP emissions are expected during the warmest seasons (the boreal and austral spring and summer). During these months, the vertical mixing in the upper layer of the ocean is reduced, leading to an increased concentration of the floating plastic at the surface.^37^ As a result, the seasonal cycle of MP emissions from the ocean surface is in antiphase with the flux of sea spray droplets (Figure 1b). Similarly, even though the Southern Hemisphere is characterized by stronger sea spray fluxes, the Northern one shows the highest MP emissions (up to 120 tons/month, reached in the month of August) while the Southern one stays below 60 tons/month, due to the lower MP mass available at the sea surface (See also the emission map in Figure S2a)

### Global Distribution of Atmospheric MP from the Oceanic Sources

**Figure 2:**
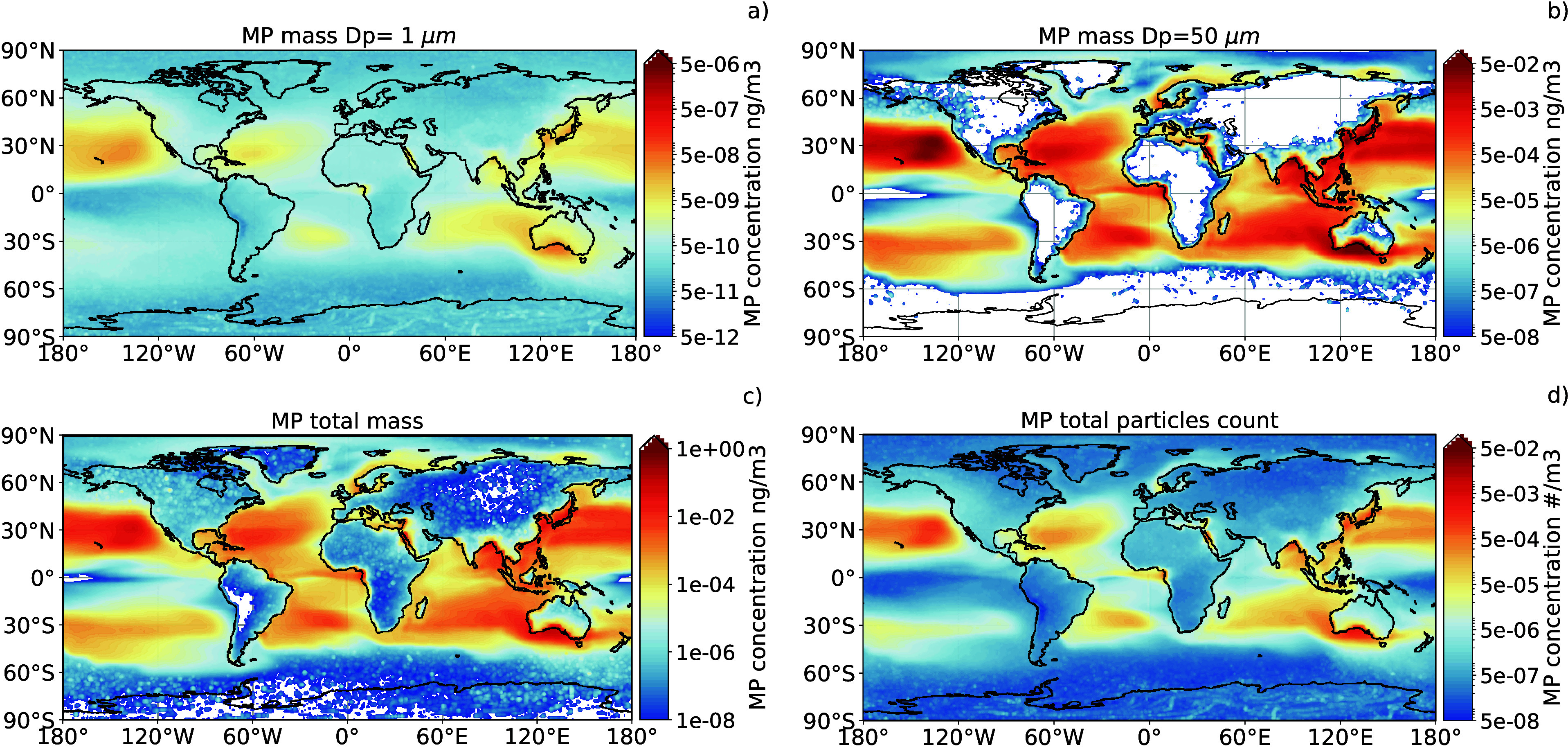
Average yearly atmospheric mass concentration in the first km of the atmosphere of oceanic MP, for Dp = 1 *μm* (panel a), Dp = 50 *μm* (panel b), all sizes with Dp ≤ 60 *μm* (panel c) and total particle number concentration for Dp ≤ 60 *μm* (panel d). Note the different scales on each map. White areas represent the absence of microplastic or concentrations below 7 orders of magnitude from the maxima. The concentrations for the other size bins are shown in Figure S3.

The resulting atmospheric MP distribution in the first km of the atmosphere is shown in Figure 2. This layer was selected as an optimal balance between having a sufficient number of Lagrangian particles in the model layer, having values representative of the order of magnitude of the surface concentration and still showing the main atmospheric transport patterns from the emissions. As expected, particles of different sizes have different atmospheric transport distributions. The larger particles, represented by the Dp = 50 *μm* class, are found mostly close to their emission regions, as a large fraction of them is quickly deposited back to the surface by gravitational settling. Despite their low residence time in the atmosphere (order of a few hours), the largest MP can reach the coastal regions of most continents (Figure 2b). In some geographical areas, in particular Southeast Asian regions and the Australian continent, the transport of oceanic MP can reach further inland. Smaller particles are transported further away from the sources and, in particular at Dp = 5 *μm* and Dp = 1 *μm*, MP particles are efficiently transported all around the globe. Except for the Dp = 50 *μm* particles, the MP seems to easily reach remote regions, including the Antarctic continent, which is far away from other possible MP sources. The distribution of values of marine MP concentration in the first km of the atmosphere, considering all the size bins used in this simulation, has a 98th percentile value of the order of 0.01 *ng* · *m*^–3^ and a maximum of 0.4 *ng* · *m*^–3^ above the oceanic MP accumulation regions (i.e., North Pacific Gyre and coastal Asian regions, Figure 2c). Figure 2d shows atmospheric MP concentrations in particles/m^3^, cumulated over the different size bins, as these are the units more often reported in cruise campaign sampling. The concentrations usually observed in these campaigns range between 10^–3^ and 10^–1^ particles per *m*^3^.^7,9,56−58^ In our simulations, if we integrate only over the size ranges tipically collected during the observations (*Dp* ≥ 10 *μm*), the concentrations in areas close to the emission regions are estimated to vary between 10^–4^ and 10^–2^ particles/*m*^3^ (up to 0.05 particles/*m*^3^ for the upper uncertainty range, see Figure S4). As highlighted in Figure 1, the intensity of the MP flux is dominated by the MP mass available from the ocean surface. That constitutes in itself a source of uncertainty, as also pointed out by Shaw et al.^34^ It is worth noting that our model starts from a total global mass of microplastic that is 1 order of magnitude above the a posteriori mass estimated in Kaandorp et al.^59^ The simulated atmospheric concentrations derived from our oceanic mass input, however, appear to be underestimated by 1 to 2 orders of magnitude compared to the atmospheric observations collected in the free ocean, where we expect the ocean contribution to be dominant compared to land, due to the proximity of the source. That implies that there may be an important underestimation in our current knowledge of the oceanic plastic pollution mass, in particular for the mass available at the surface from the particles in the micrometer range. One important source of uncertainty is related to the existence of the sea surface microlayer (the boundary interface between the atmosphere and ocean, 10^–3^ to 1 *mm* thick), where MP particles can accumulate in higher concentrations with respect to the underlying water.^60^ The enrichment factor in this layer is still not well-defined, and it has been observed to be variable, with values ranging from twice, up to hundreds of times the concentration in the underlying water.^61−63^ This can potentially explain a large part of the difference we detected between the model and the observations. Collecting information at the sea surface in this size range is still particularly challenging;^64^ however, conducting more observational campaigns targeting MP in the nano and microscale, which also bears the most significant environmental and health impacts,^65,66^ will be essential to properly constrain the presence of these particles in the marine and atmospheric environment. The possible impact of these particles on the environment may also be enhanced by the dramatic changes in the physical and chemical properties of oceanic weathered MP, including changes in morphology, electrostatic properties, hydrophobicity and sorption potential.^67^ In addition, MP in the ocean may have formed a coating of organic and biological material, which could increase their environmental risks and eco-toxicity.^68^ How these aged particles interact with the atmosphere, though, is still largely unknown.

### Deposition Fluxes at the Surface

**Figure 3:**
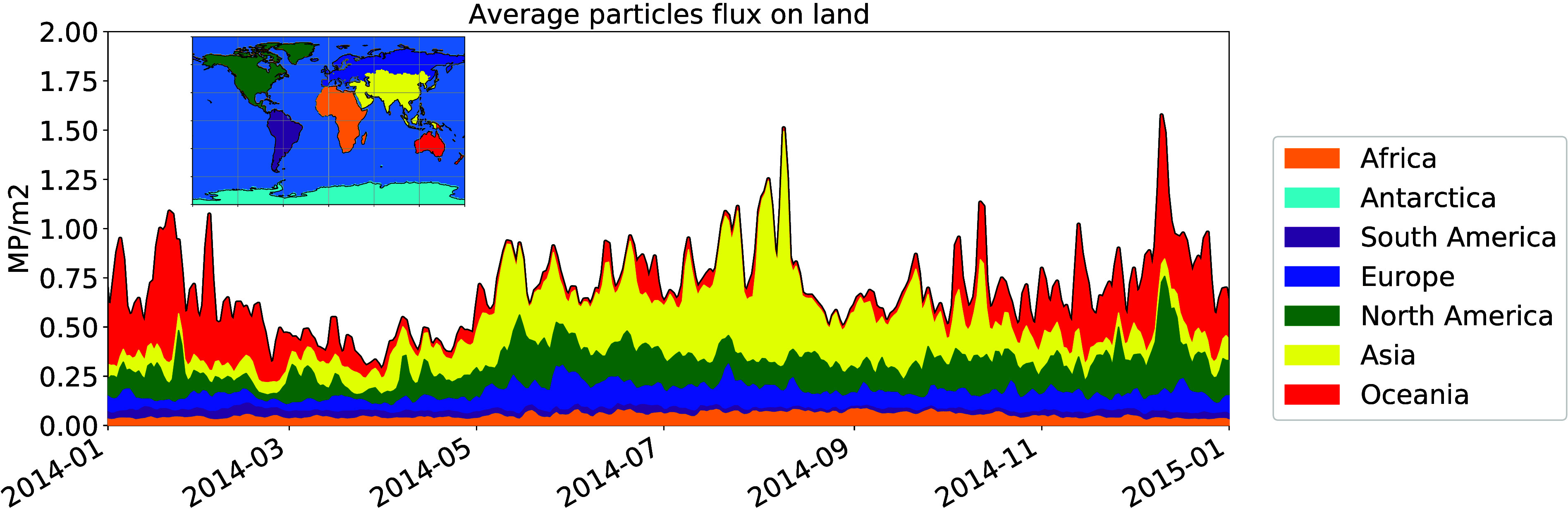
Daily variability of marine MP deposition (wet+dry deposition) flux over land, integrated over all size bins with Dp ≤ 60 *μm*. The values are averages over the surface of each continent, defined as shown in the mask in the subpanel. Antarctica and South America are not easily distinguishable in the plot as they receive little amounts of marine MP (see also Figure S6 for seasonal fluxes on each continent).

Through atmospheric transport, MP pollution from the ocean may be redistributed to the land surfaces or to the ocean itself. With our model simulations, we evaluated the deposition fluxes of the MP particles for the whole globe, including both wet and dry deposition processes. The daily variability of the average MP fluxes at the surface is shown in Figure 3 and the fluxes by size bin are shown in Figure S5. Asia and Oceania appear to be the two main continental regions affected by the influx of oceanic MP. The hemispheric seasonal pattern is clearly identifiable in the deposition fluxes on these two continents (also highlighted in Figure S6 of the SI), with the highest fluxes during the respective warm months (November to March for the Southern Hemisphere and April to October for the Northern Hemisphere). These two regions are particularly sensitive to marine MP import, due to the very high concentration of MP observed near their coasts (see Figure 2). In both regions, the MP particles of all size bins can easily reach the coast, reflecting the seasonality of the emissions in the deposition fluxes. The other continents (North and South America, Europe and Africa) have a less marked seasonal cycle in the deposition fluxes. In these regions, the dominant sizes that reach the land are smaller (Dp ≤ 10 *μm*). From the analysis of the time scales of deposition rates from the model for each size bin (not shown), it appears that the larger particles (Dp = 50 *μm* to Dp = 10 *μm*) have residence times of hours to a few days, while the smaller ones can be suspended for longer times, up to one month for Dp = 5 *μm* and two months for Dp = 1 *μm*.

**Figure 4:**
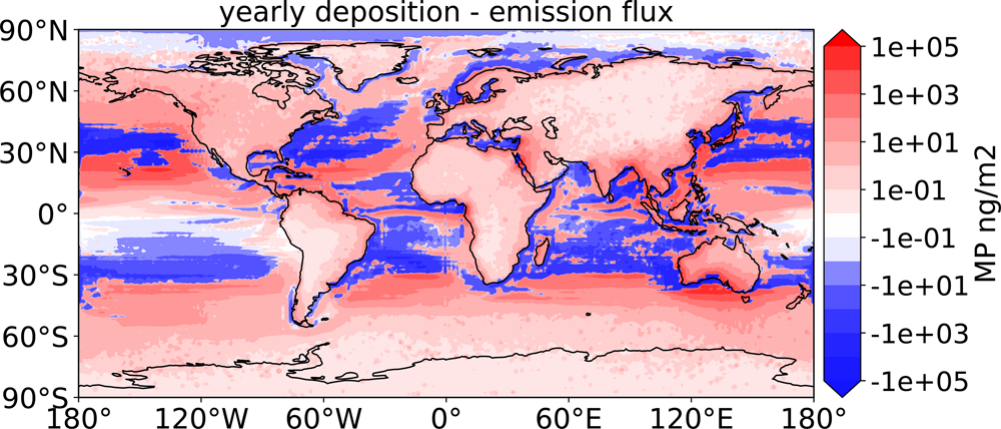
Yearly net flux of MP between the ocean surface and atmosphere. The red values represent regions with a net positive deposition flux and the blue values are the regions with emission fluxes higher than the deposition ones.

The values shown in Figure 3 represent the average over the whole surface of each continent, including all the size bins from 1 to 60 *μm*. Most of the values reported in the literature for deposition rates of atmospheric microplastic range between units to hundreds of particles per *m*^2^ per day, but include MP of larger sizes (normally above 50 *μm*,^69,70^), so a direct comparison of the values is difficult. If we only consider the particles of the upper bin size (Dp = 50 *μm*), we obtain average values over the land of the order of 10^–1^ particles per *m*^2^ (Figure S3), with maxima (not shown) reaching 10 to 50 particles per *m*^2^ close to the coasts. Even considering a possible underestimation of one to 2 orders of magnitude, it appears that the ocean sources represent a non-negligible but, overall, a nondominant source for land regions, except for the coastal areas of the most exposed regions (such as Australia, especially in the South coast, and Southeast Asia, see Figures 2 and 4) where the atmospheric import of MP from the sea can have a significant impact. A previous study Brahney et al., constraining a global atmospheric model with the Western US MP deposition data, estimated that 11% of atmospheric MP mass is coming from oceanic emissions. Our results suggest that this number is likely overestimated, in agreement with the conclusion of Fu et al.,^55^ who based their results on the assimilation of global atmospheric data.

In total, we estimate the ocean to lose, by emission of MP particles in the atmosphere, up to 19 tons of plastic per year. This represents approximately 1% of the total mass we estimate to be present on the surface for particles with Dp≤ 60 *μm*, and about 4.7 ppm of the total riverine influx of plastic (median estimate is 1.4Mt yr^–1^, see Lebreton et al.). If atmospheric emissions do not constitute a significant MP sink for the ocean, the atmospheric transport can redistribute small MP particles on the surface of the ocean (see also the comparison between the total emissions and the total deposition fluxes in Figures S2a and S2b). In particular, our simulation demonstrates the net transport of atmospheric MP from the subtropical regions, which are major oceanic accumulation zones of MP, to the higher latitudes (see Figure 4). This is particularly relevant south of the Great Pacific Garbage Patch (between 10 and 30°N) and South of Australia, where we simulated the largest positive net deposition fluxes (up to 10^5^*ng*/*m*^2^). Finally, a particularly interesting region is the Arctic, characterized by a net positive emission flux (Figure 4). This appears to be limited to the summer months (June, July and August, see Figure S8), when there is more ice-free ocean surface, while in other seasons the Arctic acts as a net receptor of marine MP transport, most likely deposited on the sea ice surface.

### Vertical Transport of MP and Pathways of Injection into the Stratosphere

**Figure 5:**
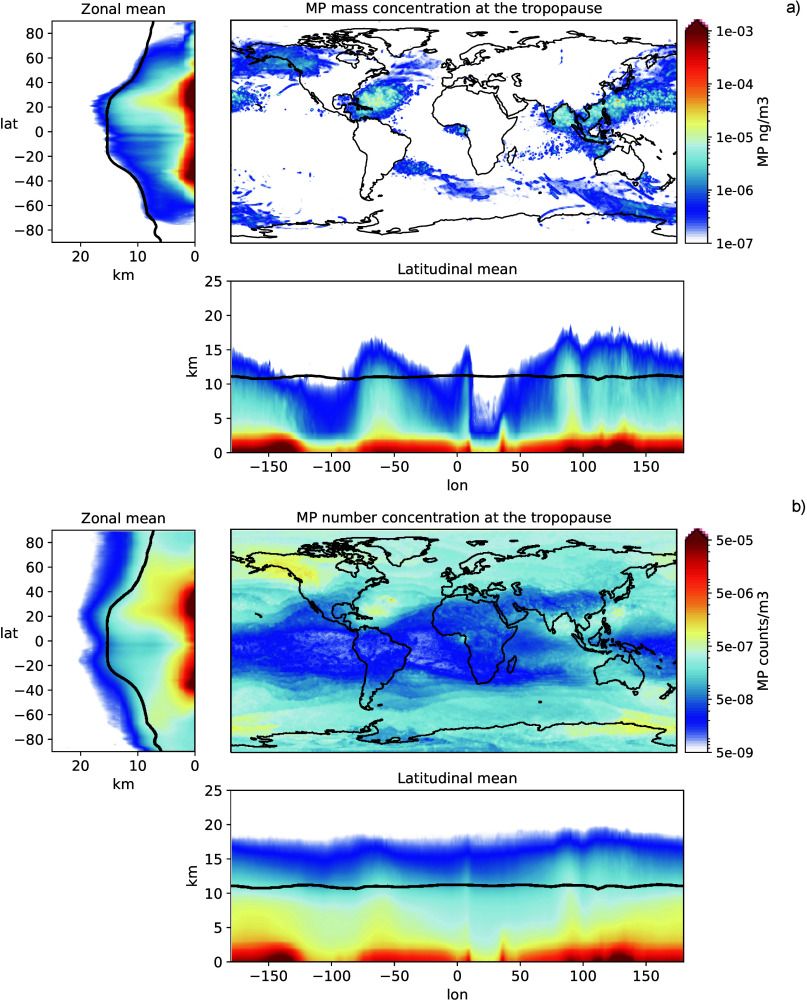
Yearly vertical transport of MP (zonal mean, latitudinal mean and concentration at the tropopause): average mass concentration (panel a) and particle number (panel b). The black line represents the average tropopause height as reported from ERA5 reanalysis.

Once MP are injected above the sea surface, they may be further uplifted and mixed in the atmosphere, and possibly interact with its physical and chemical processes. Aged MP particles, as the ones we expect to be emitted from the ocean surface, have been shown to be more hygroscopic than pristine plastic^54^ and have hence the potential to act as cloud and ice condensation nuclei.^30,32^ A recent study^71^ found evidence of MP presence in cloudwater over Japan at an altitude of 1300 to 3776 m, in the size ranges of 7 *μm* to 94 *μm*, with a concentration of 6.7 to 13.9 particles per liter (order of magnitude of 10^–4^*particles*/*m*^3^, compatible in the range of 1 order of magnitude, with the average 2.6 × 10^–5^*particles*/*m*^3^ that we obtain at the same time and location from our simulations). Their back trajectory analysis suggested that the MP were of oceanic origin. The mean vertical distribution we obtain shows indeed how the particles are transported upward from the ocean surface (Figure 5).

While most of the atmospheric MP mass from the marine sources remains within the planetary boundary layer level (2–3 km) with an average concentration of around 10^–3^ to 10^–2^*ng* · *m*^–3^, vertical MP transport may extend to the free troposphere and in some cases penetrate the stratosphere. The patterns of vertical transport are particularly noticeable in Figure 5a, where the average mass distribution is shown, mostly linked to the transport of the largest MP (Dp = 25 *μm* and 50 *μm*). These particles can reach altitudes above the tropopause level (extracted from the ERA5 data reanalysis used to drive the model), up to 20 km in some cases. The most intense vertical transport happens in the Northern Hemisphere, on the South-West seas of North America and around the Bay of Bengal and the Sea of China. This is not surprising, as these regions are characterized by intense deep convection and troposphere-stratosphere exchange events during the summer months. While most of these uplifted particles are falling downward quite rapidly (orders of hours to days), the smaller ones can stay suspended in the atmosphere for longer and keep being transported through the stratosphere. This is visible in the particle number concentration (Figure 5b) that are dominated by the smaller particles (Dp = 1 *μm* and 5 *μm*). These particles, once entering the stratosphere, keep being uplifted by the Brewer-Dobson circulation and are spread evenly around the globe. Figure S9a shows how, while the average MP mass transported to the stratosphere has a marked monthly cycle that peaks in July, the particle counts stay elevated for longer periods, between June and November, and have a less pronounced seasonal cycle. This cycle is visible independently of the scavenging properties used (see Figure S9). The scavenging properties mainly affect the total number of particles reaching the strato-sphere (in particular the smallest sizes), with differences up to 60% from the high to the low scavenging properties (see Figures S9b,c,d). This is a phenomenon that deserves to be further investigated, as the possible impacts of atmospheric MP on clouds and climate are still not fully understood.

## Data Availability

The emission fluxes produced in this study are available here: https://phaidra.univie.ac.at/o:2074060. The FLEXPART code can be freely downloaded from https://www.flexpart.eu/.

## Supporting Information Available

Section S1: Size distribution estimate for marine microplastic; Section S2: Scavenging Properties Sensitivity Study and Table 1 with the scavenging efficiencies used for the sensitivity study; Figure S1: Oceanic MP size distribution estimates; Figure S2: Yearly MP emission and deposition fluxes; Figure S3: Average MP concentration for Dp = 5 *μm*, Dp = 10 *μm*, Dp = 25 *μm*; Figure S4: Violin plot of the deposition fluxes along the longitude; Figure S5: Time series of MP deposition for each particle size; Figure S6: Seasonal variability of marine MP deposition over land; Figure S7: Relative differences in the yearly fluxes of marine MP deposition under different scavenging efficiencies; Figure S8: Seasonal net flux of MP between the ocean surface and atmosphere; Figure S9: Time series of MP mass concentration and particle concentration above tropopause for different scavenging sensitivities (PDF)
