# Supplementary material for: Exploring the Transport Path of Oceanic Microplastics in the Atmosphere
Source: Environ Sci Technol. 2024 Jul 30;58(32):14338–47. doi: 10.1021/acs.est.4c03216 (PMC11325545; doi:10.1021/acs.est.4c03216)
Supplement: Supplementary file 1 — es4c03216_si_001.pdf [file es4c03216_si_001.pdf]

Supporting Information: "Exploring the transport path of oceanic microplastics in the atmosphere"

Silvia Bucci,[*,†] Camille Richon,[‡,¶] and Lucie Bakels[†]

†*Department of Meteorology and Geophysics, University of Vienna, Universitätsring 1, Vienna, 1010, Austria*

‡*Sorbonne Universite, CNRS, IRD, MNHN, Laboratoire d'Oceanographie et du Climat: Experimentations et Approches Numeriques, Institut Pierre Simon Laplace (LOCEAN-IPSL), 75005 Paris, France*

¶*Laboratoire d'Océanographie Physique et Spatiale (LOPS), UMR 197 CNRS/IFREMER/IRD/UBO, Institut Universitaire Européen de la Mer, Plouzané, 29280, France*

E-mail: silvia.bucci@univie.ac.at

11 Pages, 9 figures, 1 table

Content:

- Section S1: Size distribution estimate for marine microplastic
- Section S2: Scavenging Properties Sensitivity Study and Table 1 with the scavenging efficiencies used for the sensitivity study.
- Figure S1: Oceanic MP size distribution estimates.

- Figure S2: Yearly MP emission and deposition fluxes
- Figure S3: Average MP concentration for Dp=5 μm,Dp=10 μm,Dp=25 μm
- Figure S4: Violin plot of the deposition fluxes along the longitude.
- Figure S5: Time series of MP deposition for each particle size
- Figure S6: Seasonal variability of marine MP deposition over land
- Figure S7: Relative differences in the yearly fluxes of marine MP deposition under different scavenging efficiencies
- Figure S8: Seasonal net flux of MP between the ocean surface and atmosphere.
- Figure S9: Time series of MP mass concentration and particle concentration above tropopause for different scavenging sensitivities
- Table 1: Scavenging efficiency parameters used for the sensitivity study

Size distribution estimate for marine microplastic

The lognormal size distribution, estimated from the number and mass load of MP from van Sebille et al. is shown in figure S1 in the blue solid line. The equation of the size distribution is a standard lognormal for which the probability of finding a certain diameter size Dp is given by:

$$f(D_p) = \frac{1}{(\sigma D_p \sqrt{2\pi})} \cdot \exp^{-\frac{(\ln D_p - \mu)}{(2\sigma^2)}} \tag{1}$$

where D_p is the particle diameter, μ represents the mean of the logarithm of the data and σ is the standard deviation of the logarithmic values. Assuming that the bigger debris (whose size distribution is represented by the lognormal distribution) will fragment into smaller ones

following a power law, we extrapolate the size distribution using the following equation:

$$N = CD_p^n \tag{2}$$

where N is the total number of particles and C is a constant computed by minimizing the distance between the power law and the log-normal distribution in the size range highlighted in the grey shade of Fig. S1. We obtain C=8.84e10 for n=-3, C=6.945e10 for n=-2.7 and C=1.19e10 for n=-3.3. The resulting distributions are shown in figure S1.

Scavenging Properties Sensitivity Study

As we expect that particles exposed to marine aging will be very hygroscopic, we chose for our simulations the same values of scavenging efficiency that are used for sulphate aerosols. Nonetheless, to take into account the possible effect of the scavenging properties on the results of this study, we also performed the simulations with three additional aerosol scavenging properties, using the same values as Evangeliou et al.. For consistency, we also kept the same naming as used in Evangeliou et al. (High, medium and low efficiency). The parameters used for the cloud condensation efficiency (CCNeff) and ice nucleation efficiency (INeff) are reported in the table below. The collection coefficient for below-cloud scavenging by rain and by snow are both assumed to be 1 for all the species, assuming the default values suggested in Grythe et al.. The results of the different efficiencies on the deposition fluxes are shown in Fig.S7, to be also compared with Fig. S2 of the SI.

Figure S6 highlights the lack of significant differences in the deposition patterns among the various scavenging values. The highest percentages of variations are observed only in the areas of low deposition flux (see Figure S2), e.g. over land and south of 60 degrees south. The difference in total deposited mass over a year is 2.547 Kg, 7.969 kg and 8.494 kg for the high, medium and low scavenging efficiency with respect to the values used for this paper (total yearly deposition of 1230175 kg). While a better understanding of the scavenging

efficiency of these particles may be relevant to properly quantify the fluxes of deposition at a local level, the current uncertainties of these properties are not significantly affecting the results of this paper.

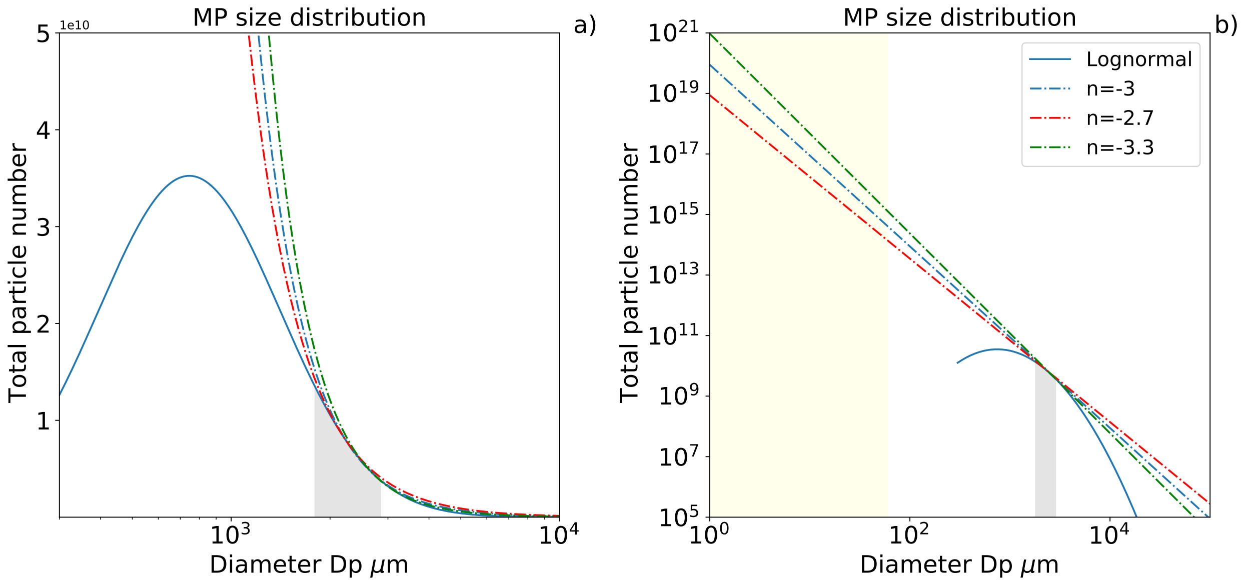

Figure S1: The solid line represents the initial size distribution, i.e. the lognormal distribution that fits the total particle numbers (51.2 trillion) and the total mass of MP (236 thousand metric tons). The dashed lines represent the estimated power law extensions ($N = C * (Dp)^n$) with 3 possible values of n: -3.3 (green), -3 (blue), -2.7 (red). C is computed by minimizing the distance between the power law and the log-normal distribution in the size range highlighted in the grey shade. For our simulations, we will use the size distribution in dashed blue as a reference, while the green and the red lines will respectively give the upper and lower estimates for the available MP mass. For a scope of clarity, we show in panel a) the lognormal distribution in linear scale zoomed on the initial size ranges, and in panel b) the lognormal distribution in logarithmic x axis with extended size ranges, to show the extrapolation down to 1 μm. The yellow shade highlights the size ranges that will be used for the atmospheric simulations, from 1 μm to 60 μm

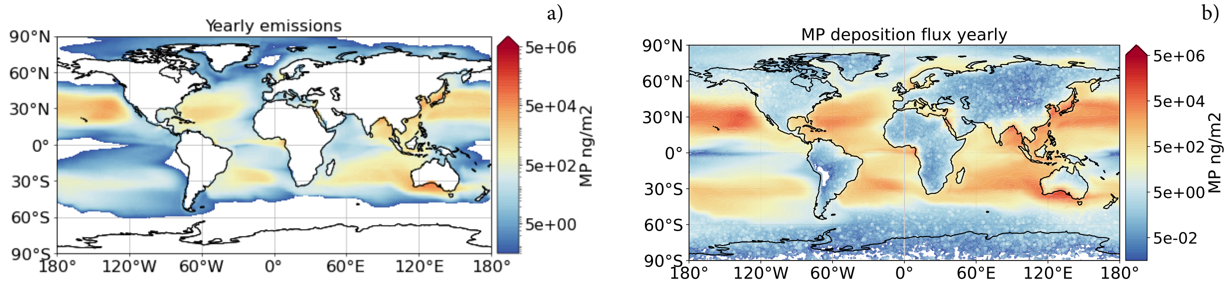

Figure S2: MP sea spray flux of mass integrated over the 1-60 μm size interval and over the whole year (panel a) and marine MP deposition flux of mass integrated over the 1-60 μm size interval and over the whole year (panel b)

Table 1: Different scavenging parameters used for the different MP species simulated in FLEXPART version 10.4. We report the different coefficients for the cloud condensation nuclei efficiency (CCNeff) and the ice nuclei efficiency (INeff) as explained in Grythe2017 et al.

**Scavenging Efficiency**	**CCNeff**	**INeff**
Used efficiency	0.9	0.1
High efficiency	0.5	0.8
Medium efficiency	0.05	0.15
Low efficiency	0.001	0.01

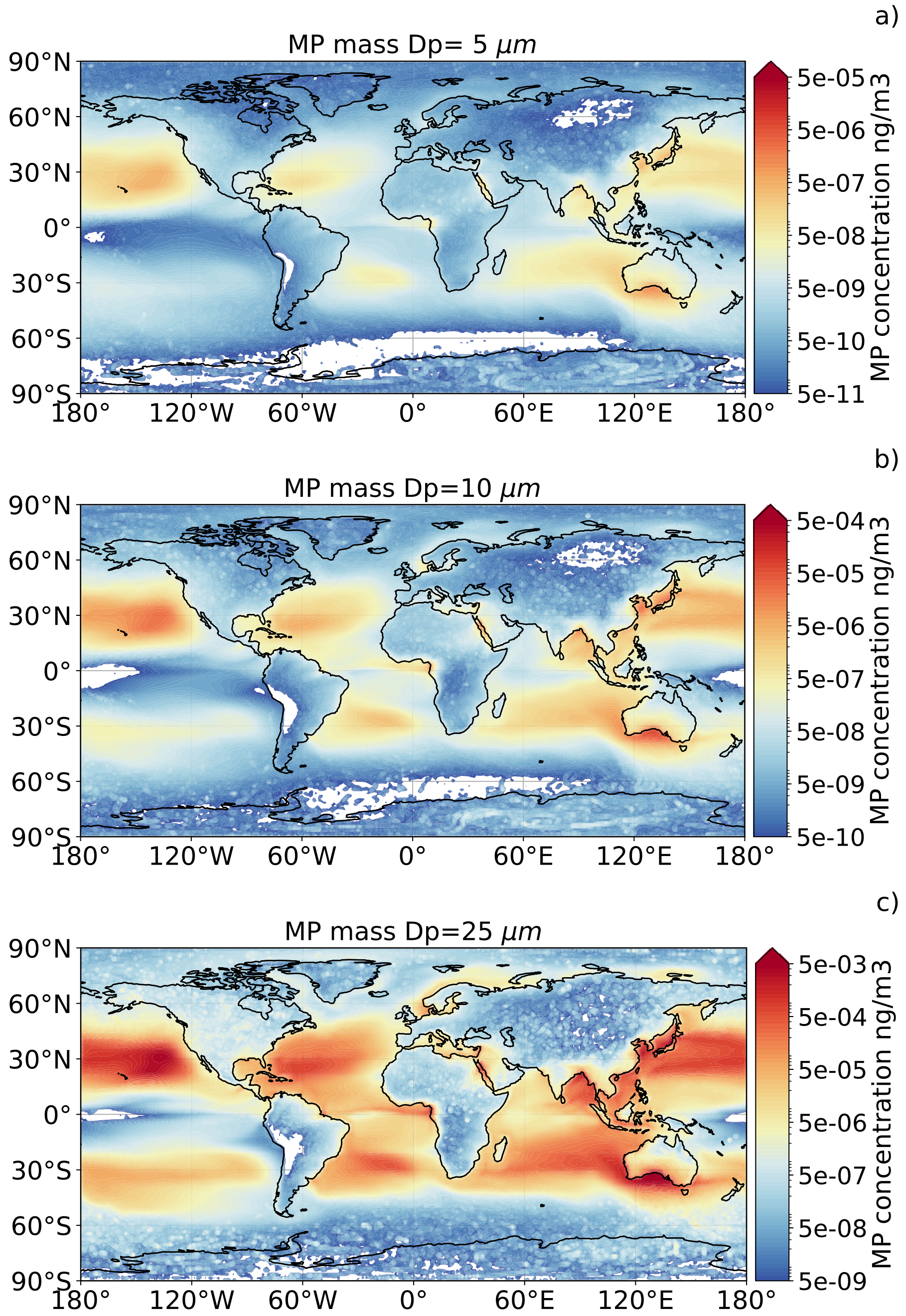

Figure S3: Average MP concentration as in Fig. 2 of the main manuscript, but for Dp=5 μm (panel a), Dp=10 μm (panel b), and Dp=25 μm (panel c)

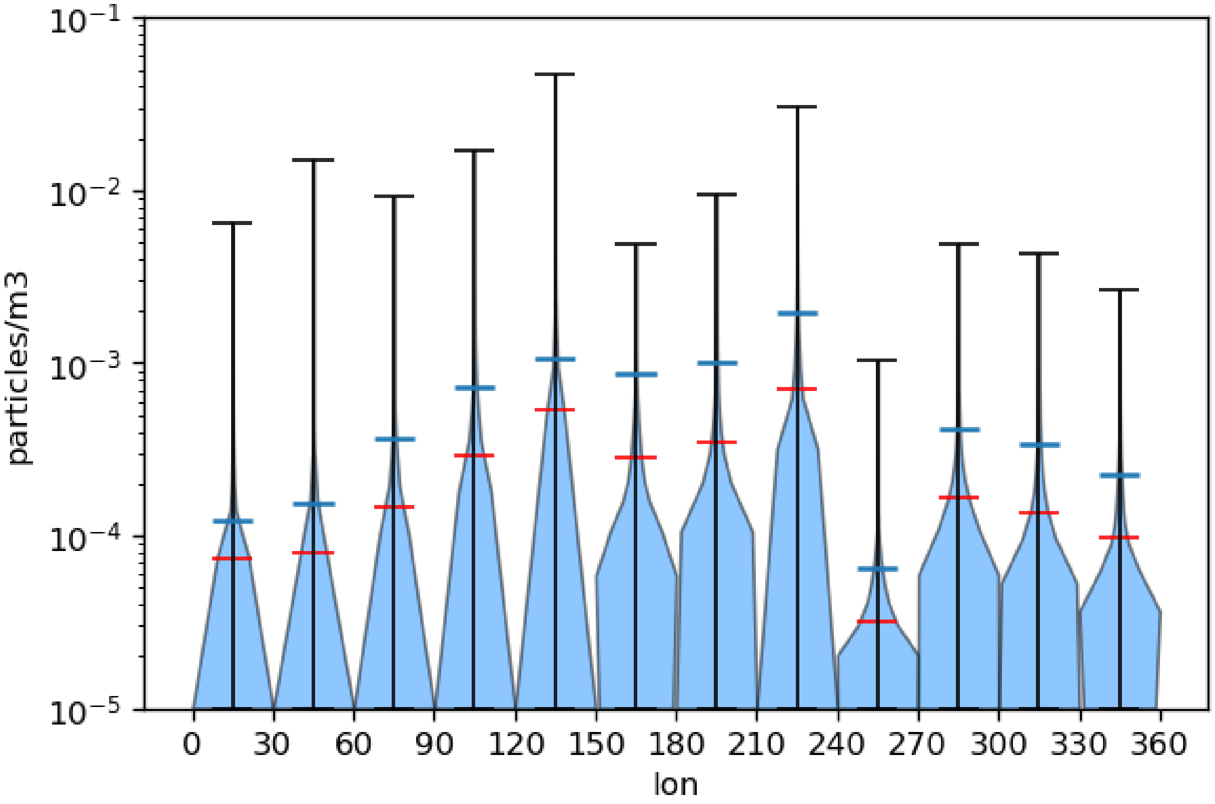

Figure S4: Violin plot of the deposition flux values every 30 degrees in longitude for particles with $Dp \geq 10\mu m$. The statistics include the estimates made with all three power law exponents (n=-3.3,n=-3,n=-2.7). The red segment represents the mean, the blue the 90% quantile, the black is the extreme values

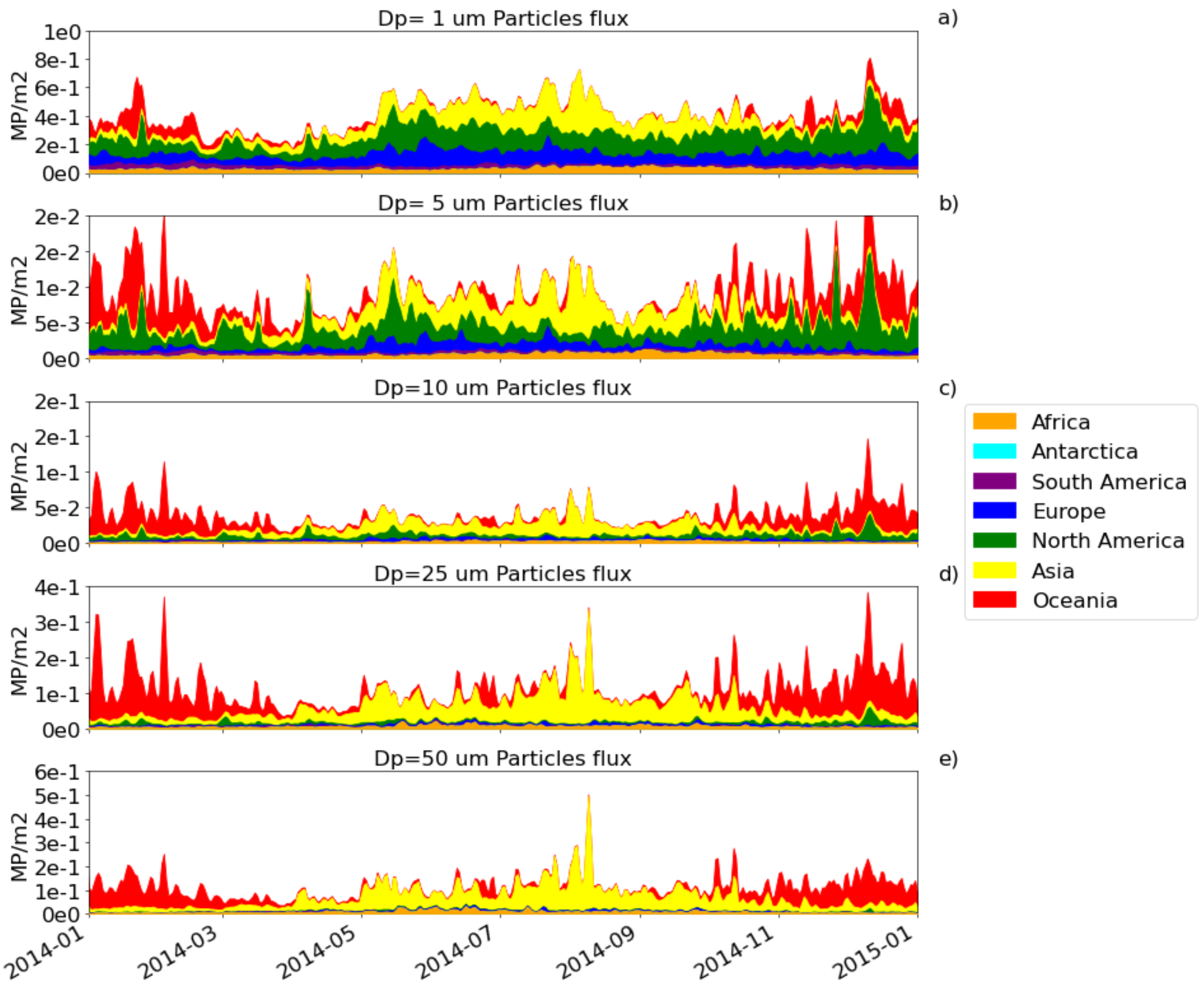

Figure S5: Daily variability of marine MP deposition (wet+dry deposition) flux over land, as in Fig 3. of the manuscript, separated for each different size bin.

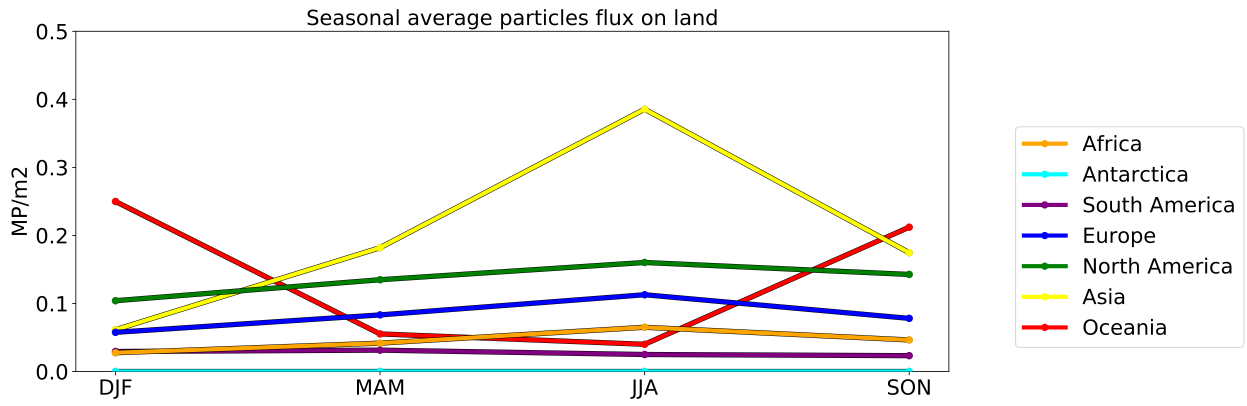

Figure S6: Seasonal variability of marine MP deposition (wet+dry deposition) flux over land, as in Fig 3. of the manuscript, integrated over December-January-February (DJF), March-April-May (MAM), June-July-August (JJA), September-October-November (SON)

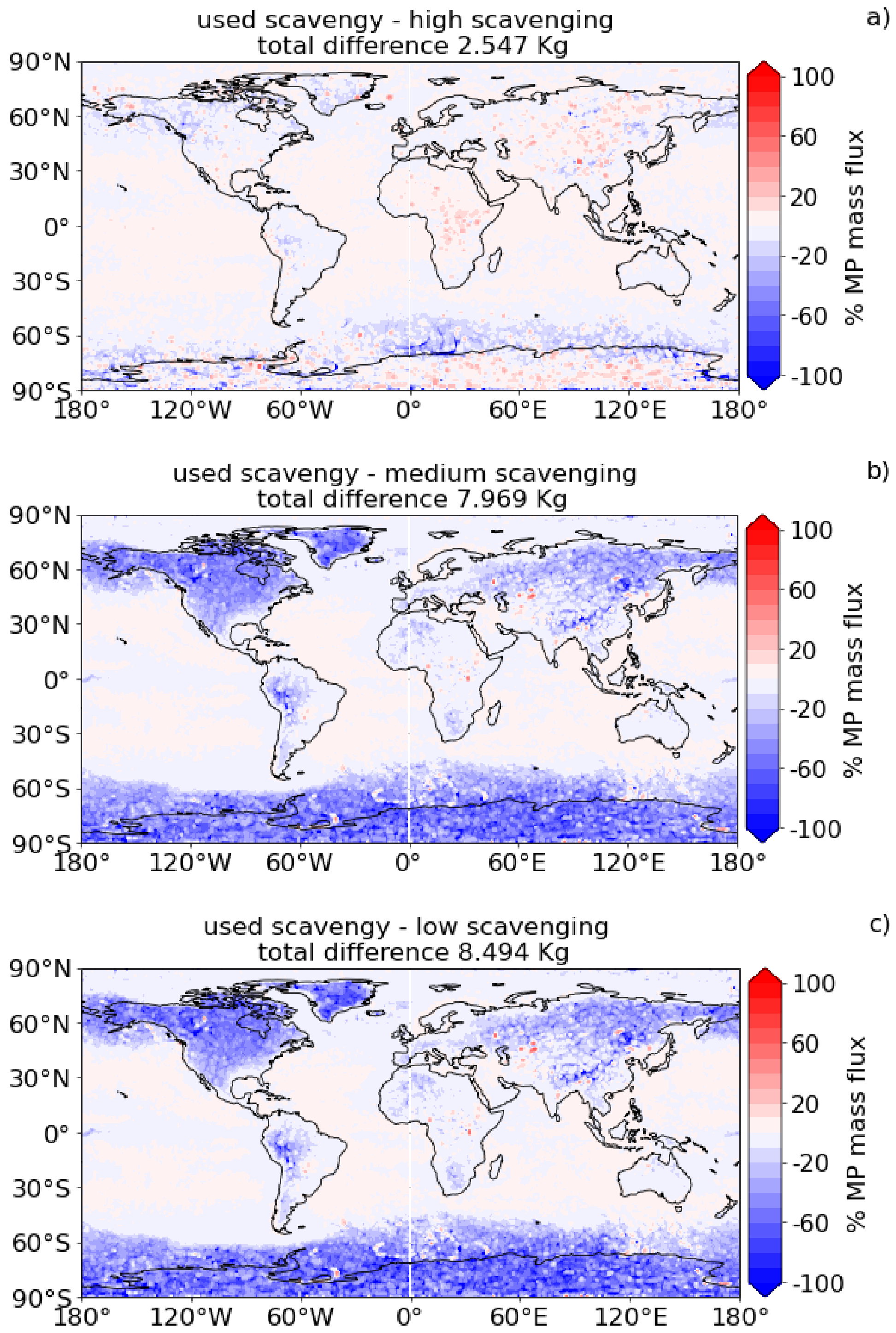

Figure S7: Relative differences in the yearly fluxes of marine MP deposition (wet+dry deposition), integrated for all size bins with Dp$\leq$60 μm, for high (panel a), medium (panel b) and low (panel c) scavenging efficiency particles, with respect to the scavenging used in the paper, as defined in Tab. 1 above. The values in the titles indicated the net difference in mass with respect to the yearly integrated deposition flux for the used scavenging efficiency

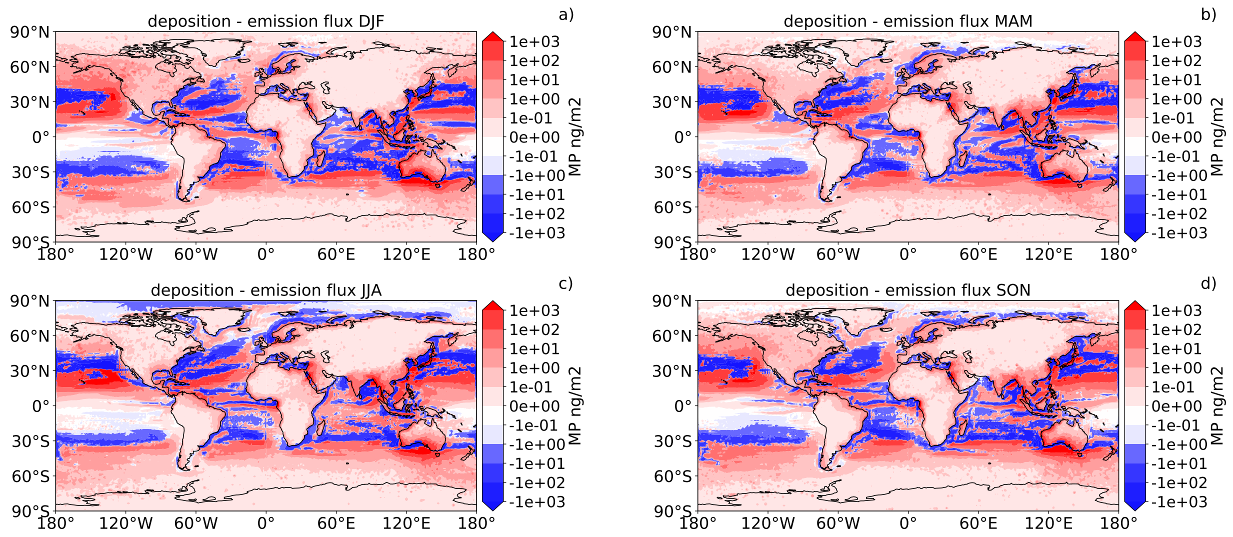

Figure S8: Seasonal net flux of MP between the ocean surface and atmosphere for December-January-February (DJF, panel a), March-April-May (MAM, panel b), June-July-August (JJA, panel c), September-October-November (SON, panel d). The red values represent regions with a net positive deposition flux and the blue values are the regions with emission fluxes higher than the deposition ones

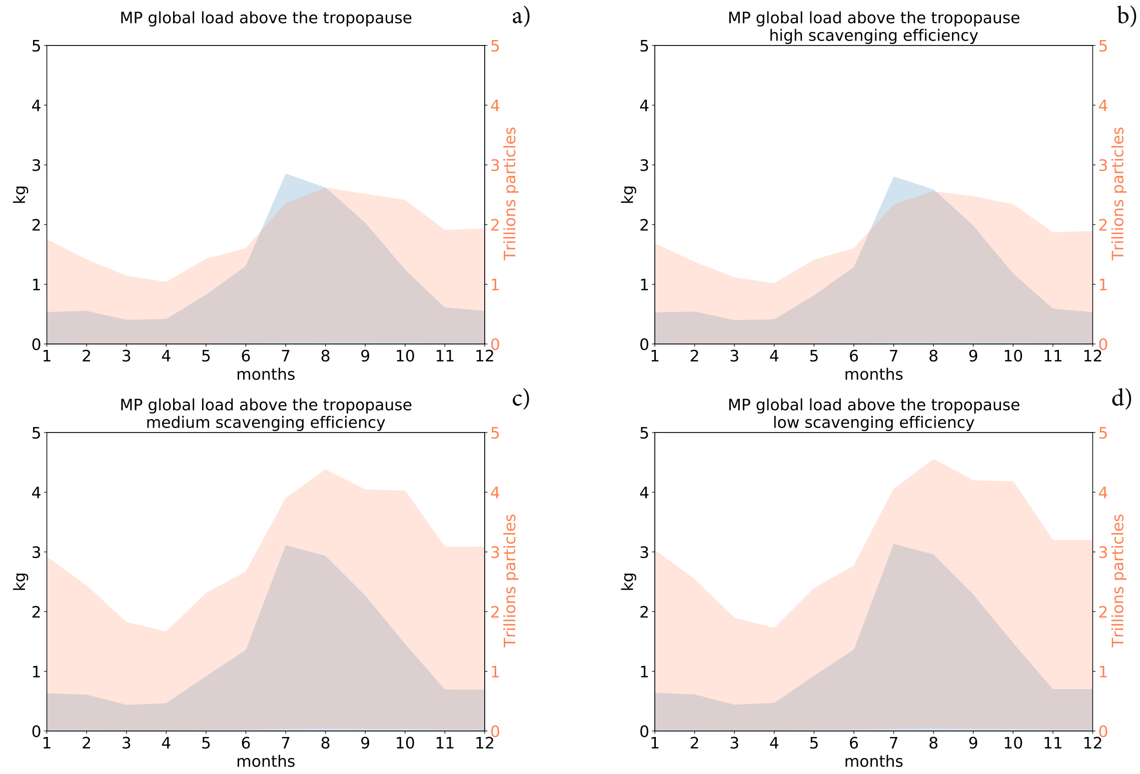

Figure S9: Global monthly mean of MP mass (blue shade) and particle count (red shade) vertically integrated from the tropopause height, as extracted from ERA5, to the highest vertical level of the model (50 km). The plot shows results using our default scavenging efficiency (panel a), which correspond to the "Used efficiency" values in Table 1 of the SI. In addition, results are shown using "High efficiency" (panel b), "Medium efficiency" (panel c) and "Low efficiency" (panel d) values from Table 1 of the SI.
